# Electroencephalographic slowing during REM sleep in older adults with subjective cognitive impairment and mild cognitive impairment

**DOI:** 10.1093/sleep/zsae051

**Published:** 2024-02-23

**Authors:** Aaron Kin Fu Lam, James Carrick, Chien-Hui Kao, Craig L Phillips, Yi Zhong Zheng, Brendon J Yee, Jong Won Kim, Ronald R Grunstein, Sharon L Naismith, Angela L D’Rozario

**Affiliations:** School of Psychology, University of Sydney, Camperdown, NSW, Australia; Woolcock Institute of Medical Research, Centre for Sleep and Chronobiology, Glebe, NSW, Australia; School of Psychological Sciences, Faculty of Medicine, Macquarie University, Sydney, NSW, Australia; School of Psychology, University of Sydney, Camperdown, NSW, Australia; Woolcock Institute of Medical Research, Centre for Sleep and Chronobiology, Glebe, NSW, Australia; School of Psychological Sciences, Faculty of Medicine, Macquarie University, Sydney, NSW, Australia; Woolcock Institute of Medical Research, Centre for Sleep and Chronobiology, Glebe, NSW, Australia; School of Psychological Sciences, Faculty of Medicine, Macquarie University, Sydney, NSW, Australia; Woolcock Institute of Medical Research, Centre for Sleep and Chronobiology, Glebe, NSW, Australia; Woolcock Institute of Medical Research, Centre for Sleep and Chronobiology, Glebe, NSW, Australia; Sydney Medical School, Faculty of Medicine and Health, Royal Prince Alfred Hospital, Camperdown, NSW, Australia; Central Clinical School, University of Sydney, Camperdown, NSW, Australia; Department of Healthcare IT, Inje University, Gimhae, Gyeongsangnam-do, South Korea; Woolcock Institute of Medical Research, Centre for Sleep and Chronobiology, Glebe, NSW, Australia; Sydney Medical School, Faculty of Medicine and Health, Royal Prince Alfred Hospital, Camperdown, NSW, Australia; School of Psychology, University of Sydney, Camperdown, NSW, Australia; Charles Perkins Centre, University of Sydney, Sydney, NSW, Australia; Woolcock Institute of Medical Research, Centre for Sleep and Chronobiology, Glebe, NSW, Australia; School of Psychological Sciences, Faculty of Medicine, Macquarie University, Sydney, NSW, Australia

**Keywords:** aging, dementia, cognition, executive function, memory, visuospatial, polysomnography, micro-architecture, power spectral analysis

## Abstract

**Study Objectives:**

In older adults with Alzheimer’s disease, slowing of electroencephalographic (EEG) activity during REM sleep has been observed. Few studies have examined EEG slowing during REM in those with mild cognitive impairment (MCI) and none have examined its relationship with cognition in this at-risk population.

**Methods:**

Two hundred and ten older adults (mean age = 67.0, *SD* = 8.2 years) underwent comprehensive neuropsychological, medical, and psychiatric assessment and overnight polysomnography. Participants were classified as subjective cognitive impairment (SCI; *n* = 75), non-amnestic MCI (naMCI, *n* = 85), and amnestic MCI (aMCI, *n* = 50). REM EEG slowing was defined as (δ + θ)/(α + σ + β) power and calculated for frontal, central, parietal, and occipital regions. Analysis of variance compared REM EEG slowing between groups. Correlations between REM EEG slowing and cognition, including learning and memory, visuospatial and executive functions, were examined within each subgroup.

**Results:**

The aMCI group had significantly greater REM EEG slowing in the parietal and occipital regions compared to the naMCI and SCI groups (partial *η*^2^ = 0.06, *p* < 0.05 and 0.06, *p* < 0.05, respectively), and greater EEG slowing in the central region compared to SCI group (partial *η*^2^ = 0.03, *p* < 0.05). Greater REM EEG slowing in parietal (*r* = −0.49) and occipital regions (*r* = −0.38 [O1/M2] and −0.33 [O2/M1]) were associated with poorer visuospatial performance in naMCI.

**Conclusions:**

REM EEG slowing may differentiate older adults with memory impairment from those without. Longitudinal studies are now warranted to examine the prognostic utility of REM EEG slowing for cognitive and dementia trajectories.

## Graphical Abstract

**Graphical Abstract:**
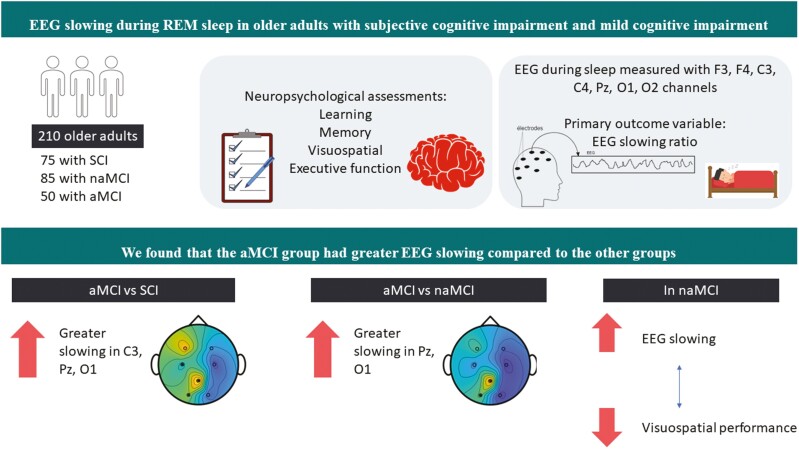

**Statement of Significance**

Using overnight polysomnography, brain activity during REM sleep was quantitatively analyzed in older adults with subjective cognitive complaints and those with amnestic and non-amnestic mild cognitive impairment (MCI). We showed greater REM EEG slowing at central, parietal, and occipital brain regions in amnestic MCI. Our findings provide evidence for slowing brain activity during REM sleep in individuals who are considered at greatest risk for developing dementia. This suggests that REM EEG slowing may be pathophysiologically linked to cognitive impairment, particularly for visuospatial impairment in individuals with cognitive impairments other than memory. Future longitudinal studies should examine the prognostic utility of REM EEG slowing for predicting cognitive decline and dementia incidence.

## Introduction

Alzheimer’s disease (AD) accounts for approximately 60%–70% of all dementia cases [1, 2]. Approximately 35% of the risk for AD is due to modifiable risk factors and the pathophysiological process of AD begins 10–20 years prior to diagnosis [3]. This has prompted efforts to examine potentially modifiable risk factors in those at a high risk of developing AD, namely those with mild cognitive impairment (MCI), where there is a 45% chance of conversion to dementia over a 5-year period. MCI is characterized by objective cognitive impairment, with generally intact functioning [4], and may be further classified as amnestic MCI (aMCI) or non-amnestic (naMCI) according to the presence or absence of memory deficits, respectively. Those with aMCI are more likely to progress to AD than naMCI, while naMCI are more likely to develop non-AD dementias such as dementia with Lewy bodies [5]. Individuals without objective cognitive deficits but self-perceived cognitive decline, often termed subjective cognitive impairment (SCI), also show an increased risk of MCI and AD [6].

Sleep disturbance is commonly reported in MCI samples compared to healthy older people. Besides having greater self-reported [7] and actigraphic [8] sleep disturbances, a recent meta-analysis of polysomnography (PSG) studies revealed that those with MCI also have prominent changes in sleep macro-architecture, including more nocturnal awakenings, less total sleep time, sleep efficiency and REM sleep, as well as increased N1 sleep, and more frequent hypoxemia events [9]. Furthermore, there is preliminary evidence for changes in sleep micro-architecture, including sleep spindle deficits during NREM sleep and abnormal EEG slowing during REM sleep in older adults with MCI [10, 11]. Abnormal REM EEG slowing is characterized by an increase in the proportion of slow-frequency brain activity relative to fast-frequency activity and has been reported in populations with neurodegenerative disease [12]. REM EEG slowing in frontal, parieto-occipital, and temporal regions has been observed in AD [12] and is predictive of cognitive impairment in patients with idiopathic REM sleep behavior disorder [13]. The only previous study examining EEG slowing in MCI demonstrated that individuals with aMCI (*n* = 22) showed greater REM EEG slowing in frontal lateral regions than those with naMCI (*n* = 10) and controls (*n* = 32) [14]. No studies have examined whether there are differences in REM EEG slowing in single and multiple-domain MCI, nor explored the potential relationship between REM EEG slowing and neuropsychological functioning.

However, prior literature has linked specific brain regions to various domains of cognitive function. For instance, EEG studies have shown that frontal brain regions are critical for executive function [15] and developmental studies have shown that maturation of central and temporal brain regions are linked to declarative memory and recall performance [16]. By contrast, EEG activity in both the parietal and occipital brain regions has been linked to visuospatial functions [17, 18]. These studies suggest a brain region-specific approach should be undertaken when investigating associations between sleep micro-architecture and cognitive functions.

The current study aimed to (1) determine whether there are differences in REM EEG slowing in the frontal, central, parietal, and occipital regions between participants with SCI, naMCI, aMCI; and (2) explore the associations between REM EEG slowing and cognitive performance in the domains of verbal learning and memory, visuospatial, and executive functioning. We hypothesized that (1) aMCI would demonstrate the greatest REM EEG slowing compared to both the naMCI and SCI groups; and (2) greater REM EEG slowing at specific brain regions would be associated with poorer executive functioning (frontal EEG slowing), learning and memory deficits (central EEG slowing) and impaired visuospatial performance (occipital and parietal EEG slowing).

## Materials and Methods

### Participants and protocol

Participants were recruited from the Healthy Brain Aging (HBA) Clinic, University of Sydney, Camperdown, Australia. The HBA clinic is a research memory clinic that provides diagnostic assessment and clinical services for older adults aged ≥50 years with new-onset cognitive and/or mood concerns referred by a GP or specialist physician. The exclusion criteria for the clinic were: dementia diagnosis or mini-mental state examination (MMSE) < 20, current or history of neurological disorder (e.g. Parkinson’s disease or epilepsy), history of head injury with a loss of consciousness greater than 30 minutes, history of stroke or transient ischemic attack, substance misuse, inadequate English skills to take part in the clinic assessments.

For the purposes of this analysis, we selected participants for this study who had subjective or objective cognitive. Participants were also excluded if they had a current diagnosis of a major depressive episode, taking medication that may affect sleep EEG physiology (e.g. imipramine, SSRIs), excessive alcohol consumption as defined by 14 standard drinks or more per week, and, did not complete PSG.

The University of Sydney Human Research Ethics committee approved this research study (Protocol No. 2012/1873), and all participants provided written informed consent.

Telephone screening was conducted for all participants for initial eligibility prior to being invited for assessment. As previously published [19], following the telephone screening, participants completed a neuropsychological, psychological, and clinical (by a geriatrician or neurologist) assessment during a daytime visit at the HBA clinic and then on a separate visit, participants underwent overnight PSG at the Woolcock Institute of Medical Research, Glebe, Sydney, Australia.

### Neuropsychological and clinical assessment

The medical assessment included a semi-structured interview that recorded a detailed medical history, including body mass index, any current medication, alcohol intake, and global cognition measured by MMSE [20]. Medical impact was measured with the Cumulative Illness Rating Scape-geriatric version (CIRS-G) [21]. The Structured Clinical Interview for the DSM-IV was used to assess current major depressive episodes [22].

As previously described [8], all participants were administered a comprehensive standardized test battery by a clinical neuropsychologist. For this sub-study, we examined:

- Learning and memory, assessed using the Rey Auditory Verbal Learning Test (RAVLT [23]). The RAVLT learning component (RAVLT trials 1–5) involved asking participants to remember a list of 15 unrelated words, this was repeated five times. For the memory component (RAVLT trial 7), participants were asked to recall the 15 words after a 20-minute delay. Learning scores ranged from 0 to 75, while memory scores ranged from 0 to 15, where higher scores indicate better performance.
- Executive function was assessed using the trail-making test [24] Part B (TMT-B) and Delis-Kaplan Executive Function System Color-Word Interference Test inhibition trial (CWIT-3 [25]). TMT-B involves connecting a series of numbered and lettered circles in alternating numerical and alphabetical order (e.g. 1-A-2-B-3-C). CWIT-3 presents color words printed in a different ink color to participants (e.g. the word “red” is printed in blue ink). They were asked to attend to the color of the ink instead of reading the word. Scores were time to completion, where higher scores on both tests indicate poorer performance.
- Visuospatial ability was assessed using Rey-Osterrieth Complex Figure test (ROCF [26]). The test comprises a complex geometric figure and asks participants to copy the figure. Scores ranged from 0 to 36, where higher scores indicate better performance.

### MCI classification

Participants were classified as having MCI following established criteria [27]. MCI was defined as ≥ 1.5 standard deviation decrement in neuropsychological test scores relative to that expected based on an individual’s age and expected level of premorbid functioning (including education, predicted IQ, and occupation) and via consensus of two neuropsychologists and a medical specialist. Participants with MCI were further classified as aMCI in the presence of an objective memory impairment (i.e. ≥ 1.5 standard deviations decrement than expected in either Rey Auditory Verbal Learning Test trial 7 or Logical Memory-II Subtest of the Wechsler Memory Scale-III [28]) with an emphasis on decrements in the delayed recall component of memory tests (i.e. not just learning deficits). Clinical information (i.e. self or informant rated memory problems) was considered in consensus ratings. Conversely, individuals were classified as naMCI if the impairment was in non-memory domains including executive functioning, visuospatial functions, processing speed, language, or learning-only deficits, with intact delayed recall.

### Self-report questionnaires

The following self-report questionnaires were collected for descriptive purposes:

Depressive symptoms were measured with the 15-item Geriatric Depression Scale (GDS-15 [29]). Scores ranged from 0 to 15, with higher scores indicating greater symptom severity.

- Subjective sleep quality was measured using the Pittsburgh Sleep Quality Index (PSQI [30]). Scores ranged from 0 to 21, where higher scores indicate poorer sleep quality.
- Insomnia symptoms were measured with the Insomnia Severity Index (ISI [31]). Scores range from 0 to 28, where higher scores indicate more severe insomnia symptoms.

### Polysomnography

During attended in-laboratory, full PSG, EEG was recorded using referential derivations at 10–20 scalp positions: F3, F4, C3, C4, O1, andO2 referenced to the contralateral mastoid, as well as midline channels Fz, Cz, Pz, Oz referenced to the average of the mastoids (M1, M2). EEG signals were sampled at 512 Hz (Sandman), 256 Hz (Profusion 4), or 200 Hz (Alice-5). PSG also included electro-oculogram (EOG), chin electromyogram (EMG), electrocardiogram (ECG), nasal airflow pressure, thoracic and abdominal respiratory effort, finger pulse oximetry, body position, and leg EMG measurements. Sleep stages and respiratory events (apneas, hypopneas, and EEG arousals) were scored according to standard American Academy of Sleep Medicine scoring criteria [32]. All participants completed their PSG within three months of completing their HBA assessment (average 22.4 days, *SD* = 18.0).

### Quantitative EEG analysis

All PSG recordings underwent automated EEG artifact detection, followed by manual visual quality checks. As previously described, a validated automated algorithm identified EEG artifacts in 5-s epochs which were subsequently rejected prior to quantitative EEG analysis [33].

We obtained EEG power spectra from F3, F4, C3, C4, O1, O2, and Pz using a fast Fourier transform routine for each consecutive 5-second epoch. Absolute spectral power was calculated within δ (0.5–4.5 Hz), θ (4.5–8 Hz), α (8–12 Hz), σ (12–15 Hz), and β (15–32 Hz) frequency ranges. The absolute EEG power for each sleep-staged 30-second epoch of the PSG recording was calculated by averaging data from up to six artifact-free 5-second epochs of EEG that comprised that 30-second recording segment. The weighted average spectral power within the defined frequency bands was then computed for REM sleep. The EEG slowing ratio during REM sleep was calculated by [(δ + θ)/ (α + σ + β)] power.

### Statistical analysis

All analyses were conducted using IBM SPSS Statistics version 26.0 (IBM Corp, Armonk, NY, USA) [34]. The normality of the distribution of dependent variables and outliers was assessed using Q-Q plots, Kolmogorov-Smirnov goodness-of-fit tests, and visual inspection of histograms. To account for non-normality, frontal, central, occipital, and parietal EEG slowing ratios were log-transformed prior to statistical analysis.

One-way analysis of variance (ANOVA) was performed to determine if there were any differences between groups (SCI, naMCI, and aMCI) on demographic characteristics. Analysis of covariance (ANCOVA) was conducted to determine whether there were differences in REM EEG slowing between the groups while controlling for age. Post hoc pairwise comparisons were conducted using Tukey HSD, where applicable to examine whether the group differences in REM EEG slowing were confounded by demographics or variables that were different between cognitive subgroups, including REM sleep duration and single or multiple domain MCI.

To assess whether EEG slowing was associated with neuropsychological measures in each cognitive subgroup, Pearson’s correlation coefficient was calculated while controlling for age, sex, and education. Based on previous literature, the following correlations were conducted: frontal REM EEG slowing ratio and executive function (TMT-B, CWIT-3); central REM EEG slowing ratio and learning and memory (RAVLT trials 1–5 and trial 7, respectively); occipital and parietal REM EEG slowing ratio and visuospatial ability (ROCF).

## Results

### Participant characteristics

Two hundred and ten participants were included in this analysis. Of these, 75 had SCI, 85 had naMCI, and 50 had aMCI. Table 1. summarizes the demographics, clinical, and self-report questionnaires for each group. Individuals with aMCI were more likely to be multiple-domain MCI compared to single-domain MCI. There were no other differences between groups on age, sex, education level, alcohol consumption, global cognition (MMSE), depressive symptoms (GDS-15), medical impact (CIRS-G), sleep quality (PSQI), or insomnia symptoms (ISI).

**Table 1.** Group Differences in Demographics, Clinical Data, and Self-report Questionnaires

	SCI *n* = 75	naMCI *n* = 85	aMCI *n* = 50	Test statistic	Effect size	*P*-value
Age, years	65.4 ± 8.9	67.6 ± 7.5	68.7 ± 9.0	2.7	0.026	0.067
Sex, male^a^ (%)	30 (40%)	31 (48%)	22 (44%)	0.7	0.060	0.688
Education, years	13.6 ± 3.0	14.0 ± 3.0	14.0 ± 3.8	0.7	0.006	0.522
BMI	28.3 ± 9.8	27.3 ± 6.4	26.2 ± 4.5	1.1	0.011	0.342
Alcohol units/week	3.97 ± 4.7	4.1 ± 4.2	2.9 ± 3.9	1.2	0.013	0.309
MMSE	29.3 ± 1.0	28.9 ± 1.3	28.2 ± 1.7	0.37	0.004	0.689
Single-domain MCI, yes (%)	—	34 (40%)^b^	10 (20%)			
Multiple domain MCI, yes (%)	—	51 (60%)^b^	40 (80%)	5.7	0.206	0.017*
GDS-15	2.8 ±** **3.2	3.3 ± 3.4	3.3 ± 3.4	0.6	0.007	0.525
CIRS-G	3.8 ± 2.9	5.4 ± 8.7	5.3 ± 3.8	2.9	0.028	0.060
PSQI	6.9 ± 3.9	7.3 ± 3.6	7.3 ± 3.7	0.4	0.004	0.647
ISI	9.3 ± 5.8	8.3 ± 5.7	10.1 ± 6.6	1.1	0.016	0.337

*p < 0.05,**p < 0.01.

Unless stated otherwise mean ± *SD* are presented. Pairwise comparisons were conducted using Tukey HSD (with *η*^2^ reported as effect size).

^a^Chi-Square test was conducted (with Phi Coefficient reported as effect size).

^b^Symbol denotes groups were not significantly different from each other.

SCI, subjective cognitive impairment; naMCI, non-amnestic mild cognitive impairment; aMCI, amnestic mild cognitive impairment; BMI, body mass index; MMSE, mini-mental state examination; MCI, mild cognitive impairment; GDS-15, Geriatric Depression Scale 15-items; CIRS, Cumulative Illness Rating Scale; PSQI, Pittsburgh Sleep Quality Index; ISI, Insomnia Severity Index.

Group comparisons on neuropsychological testing are presented in Supplementary Table S1. Given the neuropsychological testing battery was used to categorize each cognitive group, the group differences in performance were as expected. More specifically, SCI performed significantly better than naMCI and aMCI groups across all the neuropsychological tests except in verbal memory (RAVLT trial 7) where SCI and naMCI did not differ in performance. Similarly, for executive function (TMT-B and CWIT-3) SCI and aMCI also did not differ in performance. In addition, naMCI scored better than aMCI in verbal learning and memory (RAVLT trials 1–5 and RAVLT trial 7, respectively). There were no other significant pairwise differences between the groups.

### Sleep macro-architecture

The aMCI group had a shorter REM sleep duration than the SCI group but not the naMCI group, Table 2. Both the naMCI and aMCI groups had shorter N2 sleep duration than the SCI group. In addition, the EEG arousal index was higher in naMCI compared to the SCI group. The average oxygen saturation level (mean SpO_2_ %) suggested overall group differences (aMCI, naMCI, and SCI) but pairwise comparisons were not significant between groups. There were no other significant differences between groups on any other measures of sleep macro-architecture.

**Table 2.** Group Differences in Sleep Macro-Architecture

	SCI *n* = 75	naMCI *n* = 85	aMCI *n* = 50	Test statistic	Effect size (η2)	*P*-value
Time in bed, min	463.8 ± 84.9	444.6 ± 59.7	453.0 ± 57.3	1.0	0.014	0.358
Total sleep time^a^, min	365.8 ± 67.3	346.3 ± 92.1	349.5 ± 87.1	6.0	0.020	0.050
WASO ^b^, min	79.9 ± 48.9	82.2 ± 51.7	86.9 ± 50.0	0.3	0.003	0.727
Sleep efficiency, %	76.0 ± 14.7	76.0 ± 13.7	73.8 ± 12.5	0.5	0.005	0.593
Sleep latency^b^, min	25.9 ± 41.7	23.6 ± 29.8	28.2 ± 34.6	0.5	0.003	0.578
REM onset latency, min	108.0 ± 43.5	119.4 ± 63.0	110.6 ± 61.4	0.6	0.008	0.556
NREM sleep duration, min	289.4 ± 50.9	272.1 ± 57.3	281.0 ± 66.9	1.7	0.017	0.179
N1 sleep, min	23.9 ± 18.9	27.2 ± 23.4	26.9 ± 20.3	1.0	0.010	0.368
N2 sleep^c^, min	194.6 ± 50.5	166.1 ± 50.9	166.1 ± 67.2	6.4	0.059	0.002**
N3 sleep, min	73.1 ± 42.0	83.0 ± 45.7	88.4 ± 35.1	2.1	0.020	0.121
REM sleep ^a^^,^^d^, min	75.0 ± 43.0	61.5 ± 37.0	51.3 ± 40.0	14.5	0.060	0.001**
EEG arousal index^b^^,^^e^, events per hour	15.7 ± 11.0	20.1 ± 12.5	19.2 ± 13.1	3.7	0.008	0.026^*^
AHI^a^, events per hour	8.1 ± 18.8	12.4 ± 17.4	10.8 ± 14.3	6.0	0.020	0.050
3% ODI^a^, events per hour	6.9 ± 17.1	7.3 ± 14.0	6.1 ± 12.8	0.8	0.005	0.669
Mean SpO_2 %_^a^^,^^f^	95.8 ± 2.2	95.0 ± 2.0	96.0 ± 1.3	6.0	0.020	0.049^*^
Minimum SpO_2 %_^a^	88.0 ± 7.0	86.5 ± 7.3	89.0 ± 9.0	3.2	0.002	0.210

*p < 0.05, **p < 0.01.

Unless stated otherwise mean ± *SD* are presented and group comparisons performed with ANOVA (pairwise comparisons using Tukey HSD).

^a^Median ± interquartile range, Kruskal–Wallis Test (pairwise comparisons using Mann–Whitney U test with Bonferroni corrections).

^b^Raw scores were transformed with log10 for group comparisons; however, raw mean ± *SD* are presented.

^c^Pairwise comparisons for N2 duration: naMCI < SCI, aMCI < SCI.

^d^Pairwise comparisons for REM duration: aMCI < SCI.

^e^Pairwise comparisons for EEG Arousal Index: naMCI > SCI.

^f^Pairwise comparisons for Mean SpO_2_: no significant differences.

SCI, subjective cognitive impairment; naMCI, non-amnestic mild cognitive impairment; aMCI, amnestic mild cognitive impairment; WASO, wake after sleep onset; NREM, non-rapid eye movement sleep; REM, rapid eye movement sleep; AHI, apnea–hypopnea index; ODI, oxygen desaturation index; SpO_2_, oxygen saturation.

### EEG slowing during REM sleep

**Figure 1.**
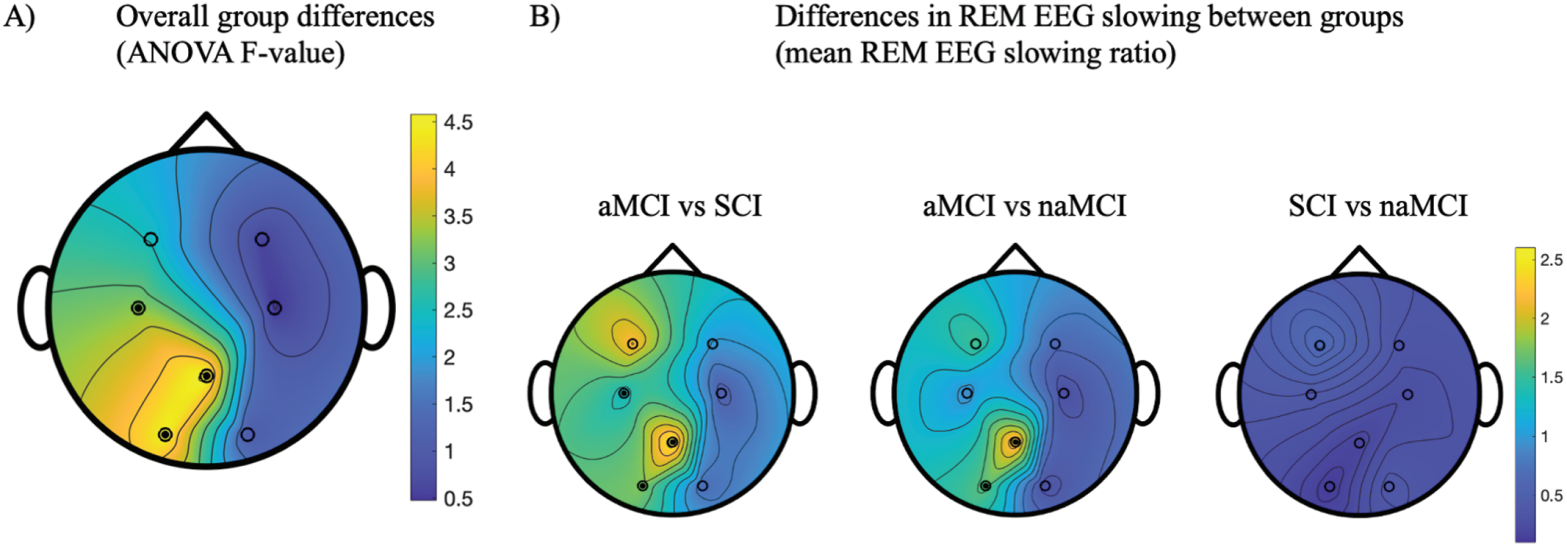
Topographical map showing the comparison between EEG slowing (in frontal, central, parietal, and occipital regions) during REM sleep across cognitive subgroups. Panel (A) Topography of ANCOVA F-value, demonstrating overall group differences in EEG slowing during REM sleep in central, parietal, and occipital regions (filled circles denote significant differences at C3, Pz, and O1 electrode sites, unfilled circles denote no significant differences). Panel (B) Topography of the pairwise difference in the REM EEG slowing ratio between groups, demonstrating (1) greater REM EEG slowing in aMCI compared to SCI in central, parietal, and occipital regions (filled circles denote significant differences at C3, Pz, and O1 electrode sites); (2) greater REM EEG slowing in aMCI compared to naMCI in parietal and occipital brain regions (filled circles denote significant differences at Pz, and O1 electrode sites); (3) no differences in REM EEG slowing between SCI and naMCI groups.

After adjusting for age, there were significant overall group differences in REM EEG slowing in central, parietal, and occipital (C3, Pz, and O1) regions between cognitive groups with small to moderate effect sizes (partial *η*^2^ = 0.033, 0.059, and 0.055, respectively), Figure 1. Pairwise comparisons revealed greater parietal and occipital REM EEG slowing in aMCI compared to both naMCI and SCI groups. While REM EEG slowing in the central region (C3) was greater in aMCI compared to SCI, it was not significantly different from the naMCI group. There were no differences in REM EEG slowing between groups for the frontal regions (F3, F4), C4, and O2. Within group REM EEG slowing ratios (mean ± *SD*) are presented in Supplementary Table S2.

### Associations between EEG slowing and cognition within groups

In participants with naMCI, there were moderate-strong correlations between visuospatial performance and parietal (Pz) and occipital (O1, O2) REM EEG slowing, after adjusting for age, sex, and education (Table 3). There were no other significant associations between REM EEG slowing and executive function (frontal REM EEG slowing), learning and memory (central REM EEG slowing), and visuospatial performance (parietal REM EEG slowing and occipital REM EEG slowing) within the groups.

**Table 3.** Correlations Between REM EEG Slowing (Frontal, Central, Occipital, and Parietal Regions) and Cognition in Older Adults With SCI, naMCI, and aMCI

		SCI		naMCI		aMCI	
Learning and memory		Pearson’s correlation	*n*	Pearson’s correlation	*n*	Pearson’s correlation	*n*
RAVLT 1–5 (learning)	C3	0.287	44	0.158	73	−0.030	48
	C4	0.156	41	0.071	72	0.087	48
RAVLT 7 (memory)	C3	0.073	44	−0.127	76	−0.039	43
	C4	−0.256	41	−0.182	75	−0.050	43
*Executive function*							
TMT-B (set-shifting)	F3	0.048	33	−0.066	68	0.173	40
	F4	0.048	40	0.073	69	0.237	44
CWIT-3 (response inhibition)	F3	−0.040	31	−0.037	66	−0.044	40
	F4	0.050	38	−0.169	67	0.121	43
*Visuospatial*							
Rey Complex Figure Test, copy	O1	−0.088	39	−0.376**	77	−0.120	37
	O2	−0.077	44	−0.329^*^	74	0.110	40
	Pz	−0.301	32	−0.490**	52	−0.115	44

*p < 0.05, **p < 0.01.

TMT-B, trail-making test part B; CWIT, color-word interference; RAVLT, Rey Auditory Verbal Learning Test; SCI, subjective cognitive impairment, naMCI, non-amnestic mild cognitive impairment; aMCI, amnestic mild cognitive impairment.

Correlations examined the relationship between REM EEG slowing in the central region and learning and memory, frontal region and executive function, and, lastly, occipital and parietal regions and visuospatial ability. O1 and Pz survives familywise Bonferroni correction (*α* = 0.013).

### Post hoc analysis

**Figure 2.**
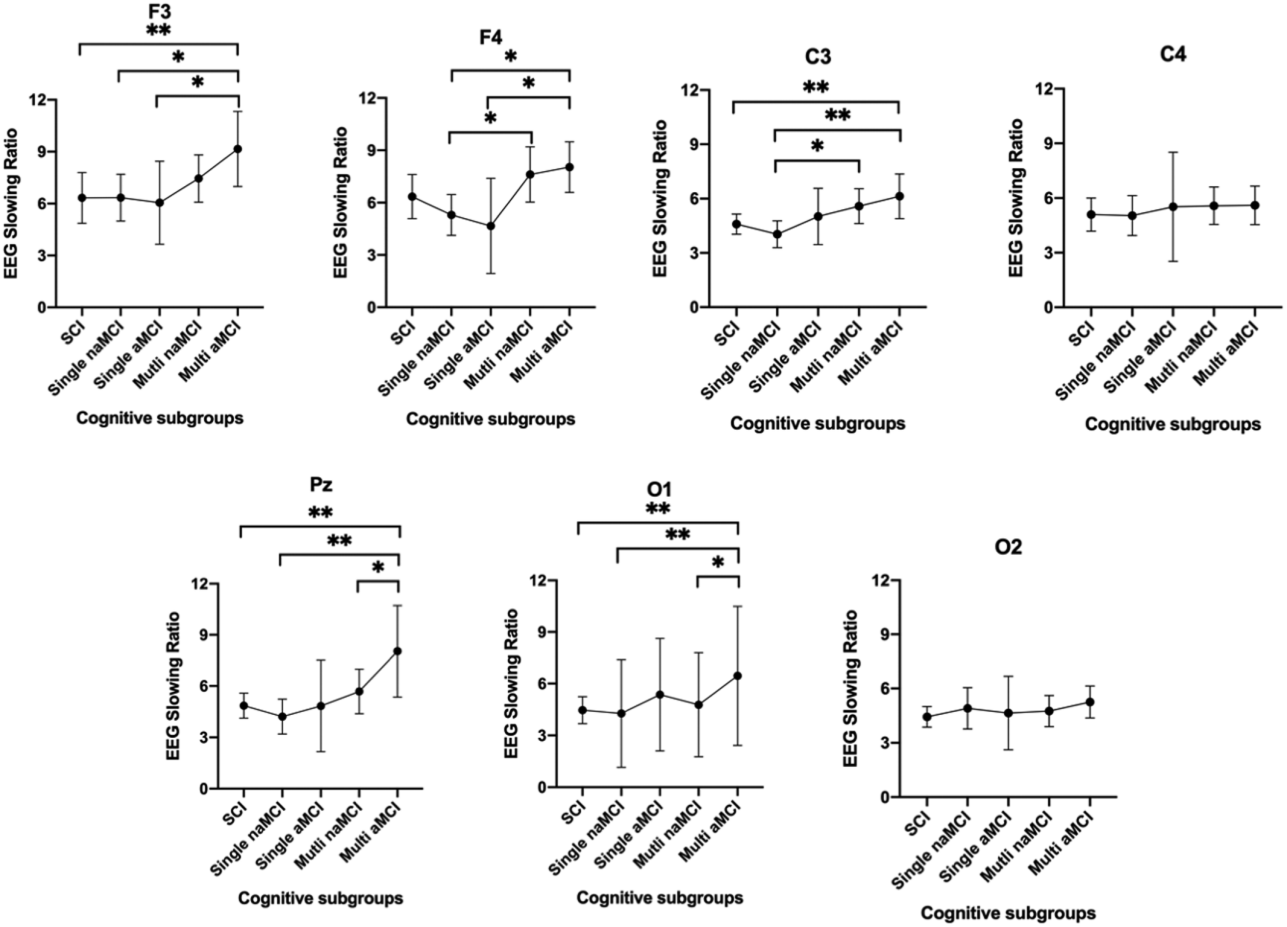
Post hoc analysis of pairwise comparisons in EEG slowing ratio during REM sleep between five cognitive subgroups to explore the impact of diffuse cognitive impairment (i.e. having multiple domain mild cognitive impairment). The five cognitive subgroups include SCI, subjective cognitive impairment; Single naMCI, single-domain non-amnestic mild cognitive impairment; single aMCI, single-domain amnestic mild cognitive impairment; Multi naMCI, multi-domain non-amnestic mild cognitive impairment; Multi aMCI, multi-domain amnestic mild cognitive impairment. For left-frontal (F3), aMCI-md had significantly more REM EEG slowing than SCI, naMCI-sd, and aMCI-sd. For right-frontal (F4), aMCI-md had significantly more REM EEG slowing than naMCI-sd and aMCI-sd. In addition, for right-frontal, naMCI-md also had greater REM EEG slowing than naMCI-sd. For right-central (C3), aMCI-md had greater REM EEG slowing than SCI and naMCI-sd, meanwhile naMCI-md also had more slowing than naMCI-sd. For medial-parietal (Pz), aMCI-md had greater slowing than SCI, naMCI-sd, and naMCI-md. For left-occipital (O1), aMCI-md had greater EEG slowing than SCI and naMCI-sd. In addition, in left-occipital, naMCI-md had more EEG slowing than naMCI-sd.

Given there were group differences in REM sleep duration, analyses were rerun with adjustment for both age and REM sleep duration. All findings remained significant after this adjustment (Supplementary Table S3). Given there was a disproportionately higher number of individuals with multiple-domain aMCI compared to single-domain aMCI, another post hoc ANCOVA (adjusting for age) was conducted to examine the group differences in REM EEG slowing between SCI, single-domain naMCI, single-domain aMCI, multiple domain naMCI, and multiple domain aMCI subgroups. Overall, there were significant group differences in F3, F4, C3, Pz, and O1. Pairwise comparisons suggest that multiple domain aMCI drives most of the overall group differences in REM EEG slowing (Figure 2). For instance, multiple-domain aMCI had significantly greater REM EEG slowing than SCI and single-domain naMCI in C3, F3, Pz, and O1 channels. Multiple-domain aMCI also had greater REM EEG slowing in Pz and O1 channels compared to multiple-domain naMCI. In participants with aMCI, those with multiple-domain aMCI also had greater REM EEG slowing in F3 and F4 compared to single-domain aMCI. In addition, multiple-domain naMCI had greater slowing in F3 and C3 than single-domain naMCI. It is important to interpret these results with caution as the sample size for each subgroup is small, in particular, the single-domain aMCI group only had 10 participants.

## Discussion

This is the largest known study to examine EEG slowing during REM sleep in older adults with MCI and in those with SCI. In 210 older adults attending a memory clinic, we observed greater REM EEG slowing in the amnestic MCI group in central, occipital, and parietal brain regions compared with the SCI group (small to moderate effect size). The aMCI group also showed REM EEG slowing in occipital and parietal regions when compared with the naMCI group (small to moderate effect size). Our study also examined the regional relationship between REM EEG slowing and cognition. Our findings partially supported our hypothesis, with a moderate association between greater REM EEG slowing in the parietal and occipital regions and poorer visuospatial performance in the naMCI group. However, REM EEG slowing was not related to any other cognitive performance measures, including executive function, learning, and memory within the cognitive groups.

In a smaller study, Brayet et al. demonstrated that aMCI (*n* = 22) had greater REM EEG slowing than naMCI (*n* = 10) and controls (*n* = 32) in the frontal lateral regions, but not in the frontal, central, occipital, or parietal regions [14]. Although our study focused on medial (not lateral) frontal regions in our MCI sample (total *n* = 135), frontal differences were not evident between any of the groups. Rather, our data showed that aMCI have EEG slowing in parietal, occipital, and central regions compared to other groups. In our post hoc exploratory analyses, we did observe greater REM EEG slowing in the frontal regions in multiple-domain aMCI compared to SCI, and single-domain naMCI and aMCI. However, by exploring single and multiple domains of MCI in this post hoc analysis, the sample size of the groups was reduced, and these results must be interpreted with caution. Nevertheless, our findings indicate that REM EEG slowing is more widespread than previously reported and not localized to the frontal lateral regions.

Given that older adults with aMCI are up to twice as likely to develop AD than naMCI [35] (those with naMCI are more likely to develop other non-AD dementias [36]), the current findings are aligned with prior work in AD samples, which also shows greater REM EEG slowing in parietal, frontal, and temporal regions compared to controls [37, 38]. Interestingly, prior work has also shown that participants with MCI with slower mean EEG frequency across all channels during REM (mean frequency was defined as the mean frequency of the averaged EEG power spectrum (4–20 Hz) a similar measure to the EEG slowing ratio) are more likely to progress to AD at 21 months follow-up [39]. Taken together, REM EEG slowing in the parietal, occipital, and central regions may suggest a greater risk of AD progression, as between-group differences were driven largely by the aMCI subtype and possibly those with multiple domain aMCI.

In this study, parietal and occipital REM EEG slowing was associated with visuospatial ability within the naMCI group. Aligned with our current findings, previous functional magnetic resonance imaging studies have shown that copying a complex figure relies on the activation of the occipital and parietal network [40]. This suggests that REM EEG slowing in the parietal and occipital regions in older adults with naMCI may map closely to visuospatial abilities and may reflect early dysfunction in the parieto-occipital network. Conversely, there were no other relationships between REM EEG slowing in any of the brain regions and learning, memory, or executive function performance within each group. This could suggest that REM EEG slowing is not sensitive to impairment in other cognitive domains or that these cognitive functions are subserved by independent brain networks. Furthermore, REM EEG slowing may represent different components of sleep micro-architecture abnormalities. For instance, delta power increases alongside a decrease in sigma may be indicative of neurological changes. However, these intricacies may not be captured by the EEG slowing ratio metric alone, as the overall ratio may not change, despite changes in brain activity within individual bands. Future longitudinal studies are necessary to examine changes in sleep micro-architecture over time and their prognostic utility for cognitive decline.

The current study suggests that EEG slowing during REM sleep can discriminate between aMCI, naMCI, and SCI groups. EEG slowing during REM sleep may indicate dysfunction in the cholinergic system. For instance, in animal models, choline acetyltransferase activity is associated with decreases in delta frequency activity and increases in theta and gamma frequency during wake and REM [41]. Loss of cholinergic neurons has been linked to memory loss, attention deficits, and reduced REM sleep duration in AD [42, 43]. Furthermore, older adults with aMCI have neurodegeneration of the cholinergic system, albeit to a lesser extent [44]. Considering the cholinergic system has extensive networks within the entire cortex, EEG slowing during REM sleep may reflect degeneration of cholinergic neurons [45], and serve as a potential tool to discriminate aMCI from other disease processes and even predict AD conversion risk. Future work would be needed to confirm whether REM EEG slowing reflects the degeneration of cholinergic neurons specifically or a more distributed network.

Our findings have several clinical implications and future research directions. Although we do not know whether REM EEG slowing reflects an underlying neuropathological process or whether it contributes to cognitive impairment and disease progression, a small randomized clinical trial demonstrated that cholinesterase inhibitor may reduce slow frequencies during REM sleep and improve cognition in older adults with mild to moderate AD [46]. Nonetheless, more research is necessitated to see if REM EEG affecting drugs may be a therapeutic intervention for at-risk groups. In addition, given the less delta power is related to greater amyloid impact [47], the effect of the recent anti-amyloid monoclonal antibodies (e.g. aducanumab) on REM EEG slowing is currently unknown.

### Strengths and limitations

The current study has several limitations worth mentioning. We were unable to assess differences in REM EEG slowing in the frontal lateral EEG region to confirm the findings of Brayet et al. [14]. Similarly, given temporal lobe atrophy may be present in the aMCI group the absence of temporal derivations in this study is a limitation worth noting. Nonetheless, our larger study showed more widespread REM EEG slowing in the parietal, occipital, and central regions. Future studies utilizing high-density EEG would provide more superior spatial resolution to assess regional EEG abnormalities during REM sleep and further explore relationships with cognitive performance. However, is it also worth noting that the PSGs derived are highly relevant and translatable for clinical practice and the findings noted herein therefore have broad clinical applicability that can be directly applied to those attending memory clinics. In addition, the study is limited by the lack of multi-model imaging. For instance, structural and spectroscopic magnetic resonance imaging may further our understanding of these EEG findings. Another limitation is the lack of AD biomarkers including β-amyloid, pathologic tau, and neurodegeneration highlighted by the research framework of the National Institute on Aging and Alzheimer’s Association [48], nonetheless, the sample is well characterized clinically utilizing a comprehensive neuropsychological test battery suitable for MCI and its subtypes and based on standardized criteria. In addition, participants with MCI are well matched in demographics and other clinical measures to their control counterparts. It is important for future work to measure longitudinal data on such samples to determine the prognostic utility of REM slowing for AD biomarker progression and cognitive decline.

## Conclusions

The current study demonstrated that EEG slowing during REM sleep is pronounced in amnestic forms of MCI, relative to both naMCI and SCI. Since older adults with aMCI are at the greatest risk of developing AD, EEG slowing during REM sleep may be clinically relevant for the early detection of AD. Therefore, measuring EEG slowing during REM sleep could indicate a different clinical trajectory that may even offer opportunities for targeted interventions.

## Supplementary Material

zsae051_suppl_Supplementary_Tables

## References

[1] Nordberg A. PET imaging of amyloid in Alzheimer’s disease. Lancet Neurol. 2004;3(9):519–527. doi: 10.1016/S1474-4422(04)00853-1 15324720

[2] Wimo A , Winblad B, Aguero-Torres H, von Strauss E. The magnitude of dementia occurrence in the world. Alzheimer Dis Assoc Disord. 2003;17(2):63–67. doi: 10.1097/00002093-200304000-00002 12794381

[3] Sperling RA , Aisen PS, Beckett LA, et al. Toward defining the preclinical stages of Alzheimer’s disease: recommendations from the National Institute on Aging-Alzheimer’s Association workgroups on diagnostic guidelines for Alzheimer’s disease. Alzheimers Dement. 2011;7(3):280–292. doi: 10.1016/j.jalz.2011.03.003 21514248 PMC3220946

[4] Knopman DS , Petersen RC. Mild cognitive impairment and mild dementia: a clinical perspective. Mayo Clin Proc. 2014;89(10):1452–1459. doi: 10.1016/j.mayocp.2014.06.019 25282431 PMC4185370

[5] Ferman TJ , Smith GE, Kantarci K, et al. Nonamnestic mild cognitive impairment progresses to dementia with Lewy bodies. Neurology. 2013;81(23):2032–2038. doi: 10.1212/01.wnl.0000436942.55281.47 24212390 PMC3854825

[6] van Harten AC , Mielke MM, Swenson-Dravis DM, et al. Subjective cognitive decline and risk of MCI: The Mayo Clinic Study of Aging. Neurology. 2018;91(4):e300–e312. doi: 10.1212/WNL.0000000000005863 29959257 PMC6070384

[7] McKinnon A , Terpening Z, Hickie IB, et al. Prevalence and predictors of poor sleep quality in mild cognitive impairment. J Geriatr Psychiatry Neurol. 2014;27(3):204–211. doi: 10.1177/0891988714527516 24687189

[8] Naismith SL , Rogers NL, Hickie IB, Mackenzie J, Norrie LM, Lewis SJ. Sleep well, think well: sleep-wake disturbance in mild cognitive impairment. J Geriatr Psychiatry Neurol. 2010;23(2):123–130. doi: 10.1177/0891988710363710 20354239

[9] D’Rozario AL , Chapman JL, Phillips CL, et al. Objective measurement of sleep in mild cognitive impairment: a systematic review and meta-analysis. Sleep Med Rev. 2020;52:101308. doi: 10.1016/j.smrv.2020.101308 32302775

[10] Gorgoni M , Lauri G, Truglia I, et al. Parietal fast sleep spindle density decrease in Alzheimer’s disease and amnesic mild cognitive impairment. Neural Plast. 2016;2016:8376108. doi: 10.1155/2016/8376108 27066274 PMC4811201

[11] Westerberg CE , Mander BA, Florczak SM, et al. Concurrent impairments in sleep and memory in amnestic mild cognitive impairment. J Int Neuropsychol Soc. 2012;18(3):490–500. doi: 10.1017/S135561771200001X 22300710 PMC3468412

[12] Petit D , Lorrain D, Gauthier S, Montplaisir J. Regional spectral analysis of the REM sleep EEG in mild to moderate Alzheimer’s disease. Neurobiol Aging. 1993;14(2):141–145. doi: 10.1016/0197-4580(93)90089-t 8487916

[13] Iranzo A , Isetta V, Molinuevo JL, et al. Electroencephalographic slowing heralds mild cognitive impairment in idiopathic REM sleep behavior disorder. Sleep Med. 2010;11(6):534–539. doi: 10.1016/j.sleep.2010.03.006 20462792

[14] Brayet P , Petit D, Frauscher B, et al. Quantitative EEG of rapid-eye-movement sleep: a marker of amnestic mild cognitive impairment. Clin EEG Neurosci. 2015;47(2):134–141. doi: 10.1177/1550059415603050 26323578

[15] Bong SH , Choi TY, Kim KM, Lee J, Kim JW. Correlation between executive function and quantitative EEG in patients with anxiety by the Research Domain Criteria (RDoC) framework. Sci Rep. 2020;10(1):18578. doi: 10.1038/s41598-020-75626-0 33122677 PMC7596478

[16] Gorgoni M , D’Atri A, Scarpelli S, Reda F, De Gennaro L. Sleep electroencephalography and brain maturation: developmental trajectories and the relation with cognitive functioning. Sleep Med. 2020;66:33–50. doi: 10.1016/j.sleep.2019.06.025 31786427

[17] Bódizs R , Lázár AS, Rigó P. Correlation of visuospatial memory ability with right parietal EEG spindling during sleep. Acta Physiol Hung. 2008;95(3):297–306. doi: 10.1556/APhysiol.95.2008.3.5 18788468

[18] Thut G , Nietzel A, Brandt SA, Pascual-Leone A. α-Band electroencephalographic activity over occipital cortex indexes visuospatial attention bias and predicts visual target detection. J Neurosci. 2006;26(37):9494–9502. doi: 10.1523/JNEUROSCI.0875-06.2006 16971533 PMC6674607

[19] Duffy SL , Lagopoulos J, Hickie IB, et al. Glutathione relates to neuropsychological functioning in mild cognitive impairment. Alzheimers Dement. 2014;10(1):67–75. doi: 10.1016/j.jalz.2013.01.005 23688577

[20] Folstein MF , Folstein SE, McHugh PR. “Mini-mental state”: a practical method for grading the cognitive state of patients for the clinician. J Psychiatr Res. 1975;12(3):189–198. doi: 10.1016/0022-3956(75)90026-6 1202204

[21] Miller MD , Paradis CF, Houck PR, et al. Rating chronic medical illness burden in geropsychiatric practice and research: application of the Cumulative Illness Rating Scale. Psychiatry Res. 1992;41(3):237–248. doi: 10.1016/0165-1781(92)90005-n 1594710

[22] Lobbestael J , Leurgans M, Arntz A. Inter-rater reliability of the Structured Clinical Interview for DSM-IV Axis I Disorders (SCID I) and Axis II Disorders (SCID II). Clin Psychol Psychother. 2011;18(1):75–79. doi: 10.1002/cpp.693 20309842

[23] Schmidt M. Rey auditory verbal learning test: A handbook. vol 17. CA: Western Psychological Services Los Angeles; 1996.

[24] Tombaugh TN. Trail Making Test A and B: normative data stratified by age and education. Arch Clin Neuropsychol. 2004;19(2):203–214. doi: 10.1016/S0887-6177(03)00039-8 15010086

[25] Delis DC , Kaplan E, Kramer JH. Delis-Kaplan executive function system. San Antonio, TX: The Psychological Corporation; 2001;

[26] Osterrieth PA. Le test de copie d’une figure complexe; contribution à l’étude de la perception et de la mémoire. [Test of copying a complex figure; contribution to the study of perception and memory.]. Arch Psychol. 1944;30:206–356.

[27] Winblad B , Palmer K, Kivipelto M, et al. Mild cognitive impairment – beyond controversies, towards a consensus: report of the International Working Group on Mild Cognitive Impairment. J Intern Med. 2004;256(3):240–246. doi: 10.1111/j.1365-2796.2004.01380.x 15324367

[28] Wechsler D. Wechsler Adult Intelligence Scale-Third Edition and Wechsler Memory Scale—Third Edition Technical Manual. San Antonio, TX: The Psychological Corporation; 1997.

[29] Sheikh JI , Yesavage JA. Geriatric Depression Scale (GDS): recent evidence and development of a shorter version. Clin Gerontol. 1986;5(1-2):165–173. doi: 10.1300/J018v05n01_09

[30] Buysse DJ , Reynolds CF, 3rd, Monk TH, Berman SR, Kupfer DJ. The Pittsburgh Sleep Quality Index: a new instrument for psychiatric practice and research. Psychiatry Res. 1989;28(2):193–213. doi: 10.1016/0165-1781(89)90047-4 2748771

[31] Bastien CH , Vallières A, Morin CM. Validation of the Insomnia Severity Index as an outcome measure for insomnia research. Sleep Med. 2001;2(4):297–307. doi: 10.1016/s1389-9457(00)00065-4 11438246

[32] Berry RB , Budhiraja R, Gottlieb DJ, et al.; American Academy of Sleep Medicine. Rules for scoring respiratory events in sleep: update of the 2007 AASM Manual for the Scoring of Sleep and Associated Events. Deliberations of the Sleep Apnea Definitions Task Force of the American Academy of Sleep Medicine. J Clin Sleep Med. 2012;8(5):597–619. doi: 10.5664/jcsm.2172 23066376 PMC3459210

[33] D’Rozario AL , Dungan GC, 2nd, Banks S, et al. An automated algorithm to identify and reject artefacts for quantitative EEG analysis during sleep in patients with sleep-disordered breathing. Sleep Breath. 2015;19(2):607–615. doi: 10.1007/s11325-014-1056-z 25225154

[34] IBM SPSS Statistics for Windows. Armonk, NY: IBM Corp; 2019.

[35] Fischer P , Jungwirth S, Zehetmayer S, et al. Conversion from subtypes of mild cognitive impairment to Alzheimer dementia. Neurology. 2007;68(4):288–291. doi: 10.1212/01.wnl.0000252358.03285.9d 17242334

[36] Boeve BF. Mild cognitive impairment associated with underlying Alzheimer’s disease versus Lewy body disease. Parkinsonism Relat Disord. 2012;18:S41–S44. doi: 10.1016/S1353-8020(11)70015-3 22166451

[37] Petit D , Montplaisir J, Lorrain D, Gauthier S. Spectral analysis of the rapid eye movement sleep electroencephalogram in right and left temporal regions: a biological marker of Alzheimer’s disease. Ann Neurol. 1992;32(2):172–176. doi: 10.1002/ana.410320208 1510357

[38] Hassainia F , Petit D, Nielsen T, Gauthier S, Montplaisir J. Quantitative EEG and statistical mapping of wakefulness and REM sleep in the evaluation of mild to moderate Alzheimer’s disease. Eur Neurol. 1997;37(4):219–224. doi: 10.1159/000117446 9208261

[39] Jelic V , Johansson SE, Almkvist O, et al. Quantitative electroencephalography in mild cognitive impairment: longitudinal changes and possible prediction of Alzheimer’s disease. Neurobiol Aging. 2000;21(4):533–540. doi: 10.1016/s0197-4580(00)00153-6 10924766

[40] Yuan Y , Brown S. Drawing and writing: an ALE meta-analysis of sensorimotor activations. Brain Cogn. 2015;98:15–26. doi: 10.1016/j.bandc.2015.05.004 26051526

[41] Lee MG , Hassani OK, Alonso A, Jones BE. Cholinergic basal forebrain neurons burst with theta during waking and paradoxical sleep. J Neurosci. 2005;25(17):4365–4369. doi: 10.1523/JNEUROSCI.0178-05.2005 15858062 PMC6725118

[42] Ferreira-Vieira TH , Guimaraes IM, Silva FR, Ribeiro FM. Alzheimer’s disease: targeting the Cholinergic System. Curr Neuropharmacol. 2016;14(1):101–115. doi: 10.2174/1570159x13666150716165726 26813123 PMC4787279

[43] Angevaare MJ , Vonk JMJ, Bertola L, et al. Predictors of incident mild cognitive impairment and its course in a diverse community-based population. Neurology. 2022;98(1):e15–e26. doi: 10.1212/WNL.0000000000013017 34853178 PMC8726570

[44] Teipel SJ , Meindl T, Grinberg L, et al. The cholinergic system in mild cognitive impairment and Alzheimer’s disease: an in vivo MRI and DTI study. Hum Brain Mapp. 2011;32(9):1349–1362. doi: 10.1002/hbm.21111 20672311 PMC5899896

[45] Mesulam MM. Cholinergic circuitry of the human nucleus basalis and its fate in Alzheimer’s disease. J Comp Neurol. 2013;521(18):4124–4144. doi: 10.1002/cne.23415 23852922 PMC4175400

[46] dos Santos Moraes WA , Poyares DR, Guilleminault C, Ramos LR, Bertolucci PHF, Tufik S. The effect of donepezil on sleep and REM Sleep EEG in patients with Alzheimer disease: a double-blind placebo-controlled study. Sleep. 2006;29(2):199–205. doi: 10.1093/sleep/29.2.199 16494088

[47] André C , Champetier P, Rehel S, et al.; Medit-Ageing Research Group. Rapid eye movement sleep, neurodegeneration, and amyloid deposition in aging. Ann Neurol. 2023;93(5):979–990. doi: 10.1002/ana.26604 36641644

[48] Jack CR Jr. , Bennett DA, Blennow K, et al.; Contributors. NIA-AA research framework: toward a biological definition of Alzheimer’s disease. Alzheimers Dement. 2018;14(4):535–562. doi: 10.1016/j.jalz.2018.02.018 29653606 PMC5958625

