# Supplementary material for: Electroencephalographic slowing during REM sleep in older adults with subjective cognitive impairment and mild cognitive impairment
Source: Sleep. 2024 Feb 23;47(6):zsae051. doi: 10.1093/sleep/zsae051 (PMC11168761; doi:10.1093/sleep/zsae051)
Supplement: zsae051_suppl_Supplementary_Tables [file zsae051_suppl_supplementary_tables.docx]

**Supplementary Materials**

**EEG slowing during REM sleep in older adults with subjective cognitive impairment and mild cognitive impairment**

Authors: Aaron Kin Fu Lam^1,2,3^*, James Carrick^1^*, Chien-Hui Kao^2,3^, Craig L Phillips^2,3^, Yi Zhong Zheng^2^, Brendon J Yee^2,5,7^, Jong Won Kim^4^, Ronald R. Grunstein^2,5^, Sharon L. Naismith^1,6†^, Angela L. D’Rozario^2,3†^

*Joint first authors.

^†^Joint last authors

^1^The University of Sydney, School of Psychology, Camperdown, NSW, AU

^2^Woolcock Institute of Medical Research, Centre for Sleep and Chronobiology, Glebe, NSW, AU

^3^Macquarie University, School of Psychological Sciences, Faculty of Medicine, Sydney, NSW, AU

^4^Inje University, Department of Healthcare IT, Gimhae, Gyeongsangnam-do, KR

^5^Royal Prince Alfred Hospital, Sydney Medical School, Faculty of Medicine and Health
Camperdown, AU

^6^The University of Sydney, Charles Perkins Centre, Sydney, NSW, AU

^7^Central Clinical School, University of Sydney, NSW, AU

Corresponding author:

Aaron Kin Fu Lam

Woolcock Institute of Medical Research

431 Glebe Point Road, Glebe, NSW 2050

[aaron.lam@sydney.edu.au](mailto:aaron.lam@sydney.edu.au)

*Supplementary Table 1. Group comparisons of neuropsychological testing performance between SCI, naMCI, and aMCI groups.*

|  | SCI  n=75 | naMCI  n=85 | aMCI  n=50 | Test statistic | Partial η2 | p-value |
| --- | --- | --- | --- | --- | --- | --- |
| RAVLT 1-5 (learning) | 39.7 ± 21.8 | 37.4 ± 19.4 | 30.5 ± 17.2 | 1.9 | 0.023 | 0.148 |
| RAVLT 7 (memory) | 11.6^a^ ± 2.9 | 10.4^a^ ± 3.2 | 5.6 ± 4.7 | 36.4 | 0.312 | 0.001** |
| TMT-B (set-shifting) | 62.5 ± 29.9 | 81.2^a^ ± 48.7 | 81.6^a^ ± 46.0 | 4.7 | 0.047 | 0.011* |
| CWIT-3 (response inhibition) | 58.9 ± 10.7 | 67.3^a^ ± 19.4 | 66.2^a^ ± 18.9 | 4.7 | 0.051 | 0.010* |
| ROCF, copy | 33.0 ± 2.7 | 29.1^a^ ± 3.8 | 29.9^a^ ± 4.7 | 12.0 | 0.156 | 0.001** |
| *p<0.05,**p<0.01.  Mean ± sd are presented. Group comparisons were conducted using an ANCOVA with Tukey HSD, controlling for age, sex and, education.  ^a^Denotes groups were not statistically significantly different on pairwise comparisons.  SCI = subjective cognitive impairment; naMCI = non-amnestic mild cognitive impairment; aMCI = amnestic mild cognitive impairment; RAVLT = Rey Auditory Verbal Learning Test; TMT-B = Trail Making Test-Part B; CWIT-3 = Color Word Interference Test Trial 3; ROCF = Rey-Osterrieth Complex Figure | | | | | | |

|  | SCI  n=75 | naMCI  n=85 | aMCI  n=50 | Test statistic | Partial η2 | p-value |
| --- | --- | --- | --- | --- | --- | --- |
| F3 | 6.3 ± 4.1 | 7.0 ± 4.1 | 8.5 ± 5.7 | 2.5 | 0.034 | 0.089 |
| F4 | 6.3 ± 4.0 | 6.7 ± 4.5 | 7.4 ± 4.3 | 0.6 | 0.008 | 0.563 |
| C3 | 4.6 ± 2.3 | 5.0 ± 2.9 | 5.9 ± 3.5 | 3.3 | 0.033 | 0.040* |
| C4 | 5.1 ± 3.1 | 5.3 ± 3.2 | 5.6 ± 3.1 | 0.5 | 0.006 | 0.624 |
| Pz | 4.9 ± 2.7 | 5.0 ± 3.1 | 7.4 ± 6.9 | 4.6 | 0.059 | 0.012* |
| O1 | 4.5 ± 2.4 | 5.6 ± 3.0 | 6.2 ± 3.9 | 4.5 | 0.055 | 0.013* |
| O2 | 4.4 ± 2.4 | 4.8 ± 3.0 | 5.1 ± 2.8 | 1.0 | 0.011 | 0.352 |
| *p<0.05,**p<0.01.  Mean ± sd are presented. Group comparisons were conducted using an ANCOVA (adjusted for age).  SCI = subjective cognitive impairment; naMCI = non-amnestic mild cognitive impairment; aMCI = amnestic mild cognitive impairment | | | | | |  |

*Supplementary Table 2. Group comparisons of EEG slowing during REM for each channel for SCI, naMCI, and aMCI groups.*

| *Supplementary Table 3. ANCOVA results controlling for age and REM sleep duration* | | | | | | |
| --- | --- | --- | --- | --- | --- | --- |
|  | SCI  n=75 | naMCI  n=85 | aMCI  n=50 | Test statistic | Partial η2 | p-value |
| F3 | 6.3 ± 4.1 | 7.0 ± 4.1 | 8.5 ± 5.7 | 2.5 | 0.035 | 0.089 |
| F4 | 6.3 ± 4.0 | 6.7 ± 4.5 | 7.4 ± 4.3 | 0.8 | 0.011 | 0.445 |
| C3 | 4.6 ± 2.3 | 5.0 ± 2.9 | 5.9 ± 3.5 | 3.6 | 0.037 | 0.030* |
| C4 | 5.1 ± 3.1 | 5.3 ± 3.2 | 5.6 ± 3.1 | 0.7 | 0.009 | 0.503 |
| Pz | 4.9 ± 2.7 | 5.0 ± 3.1 | 7.4 ± 6.9 | 3.6 | 0.048 | 0.029* |
| O1 | 4.5 ± 2.4 | 5.6 ± 3.0 | 6.2 ± 3.9 | 4.0 | 0.051 | 0.020* |
| O2 | 4.4 ± 2.4 | 4.8 ± 3.0 | 5.1 ± 2.8 | 0.9 | 0.010 | 0.394 |
| *p<0.05, **p<0.01  ANCOVA = Analysis of covariance; REM = rapid eye movement; SCI = subjective cognitive impairment; naMCI = non-amnestic mild cognitive impairment; aMCI = amnestic mild cognitive impairment. | | | | | | |
